# Supplementary material for: Foredune erosion, overtopping and destruction in 2022 at Bengello Beach, southeastern Australia
Source: Camb Prism Coast Futur. 2024 Apr 26;2:e7. doi: 10.1017/cft.2024.8 (PMC12337608; doi:10.1017/cft.2024.8)
Supplement: Oliver et al. supplementary material [file S2754720524000088sup001.docx]

Supplementary information


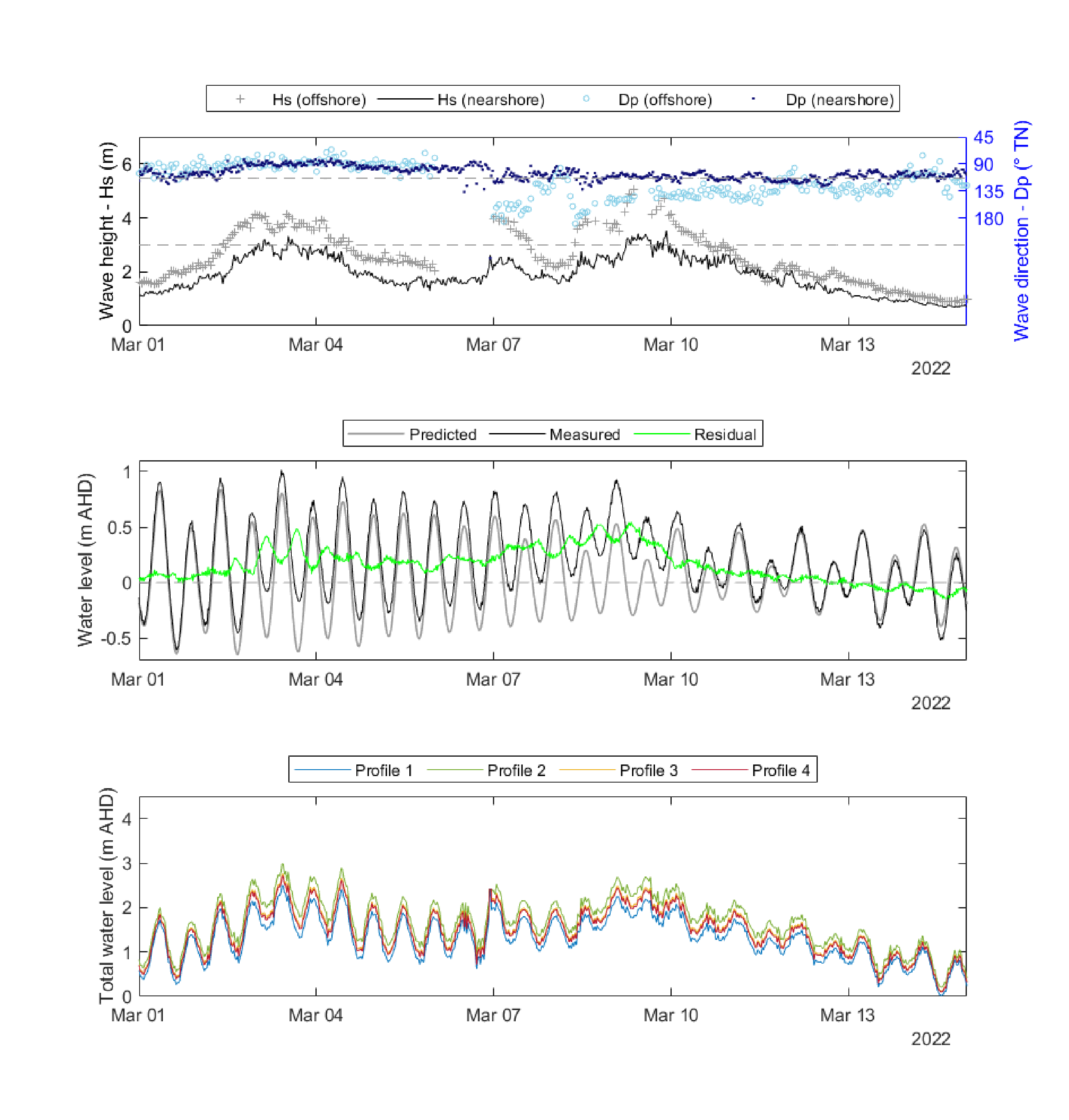


Supplementary Figure 1: Storm events of early March (Storm 1 and 2) showing wave height (Hs = H_sig_) and direction perpendicular to Bengello Beach for the offshore wave buoy and the nearshore wave buoy at Bengello Beach. Water level data (predicted, measured and residual between these) from the same period is recorded by the nearby Princess Jetty tide gauge in Batemans Bay which represents the ocean tide with the additional influence of river floods. Note the elevated residuals indicate flooding of the Clyde River. Total Water Level are shown for each of the four profiles.


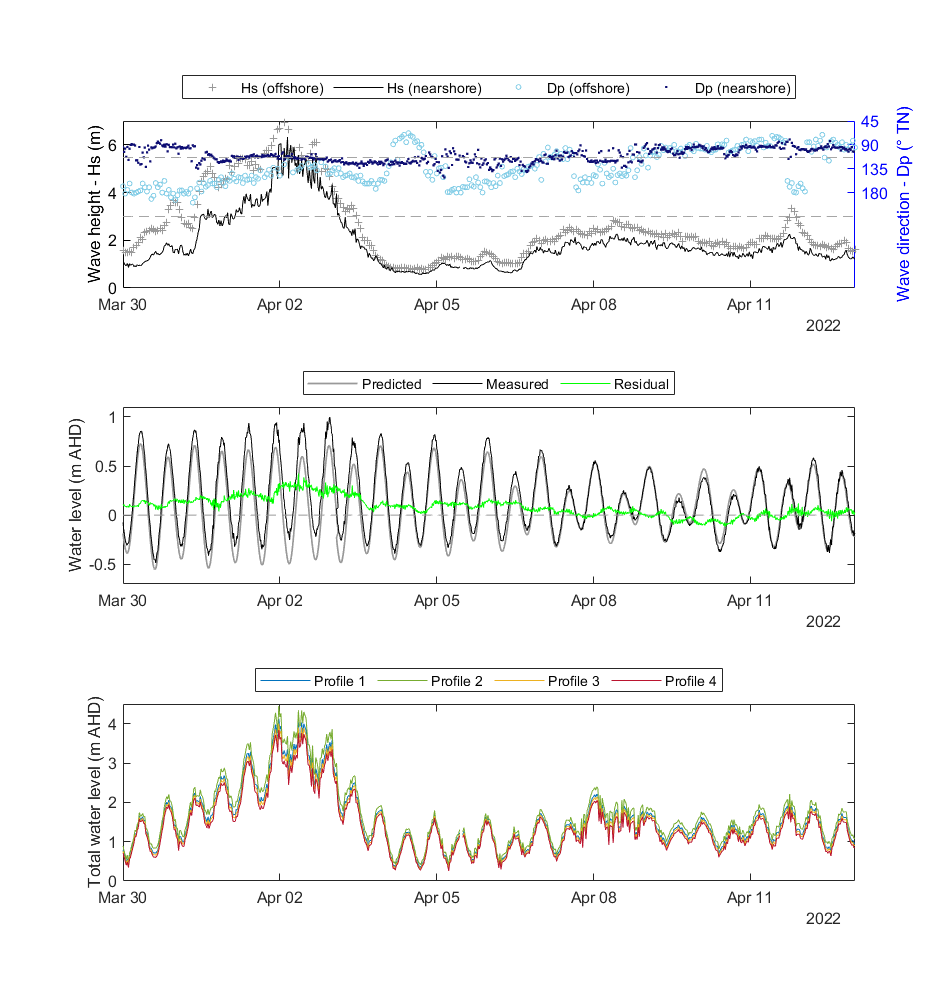


Supplementary Figure 2: Storm event of early April showing wave height (Hs = H_sig_) and direction perpendicular to Bengello Beach for the offshore wave buoy and the nearshore wave buoy at Bengello Beach. Water level data (predicted, measured and residual between these) from the same period is recorded by the nearby Princess Jetty tide gauge in Batemans Bay which represents the ocean tide with the additional influence of river floods. Total Water Level are shown for each of the four profiles.


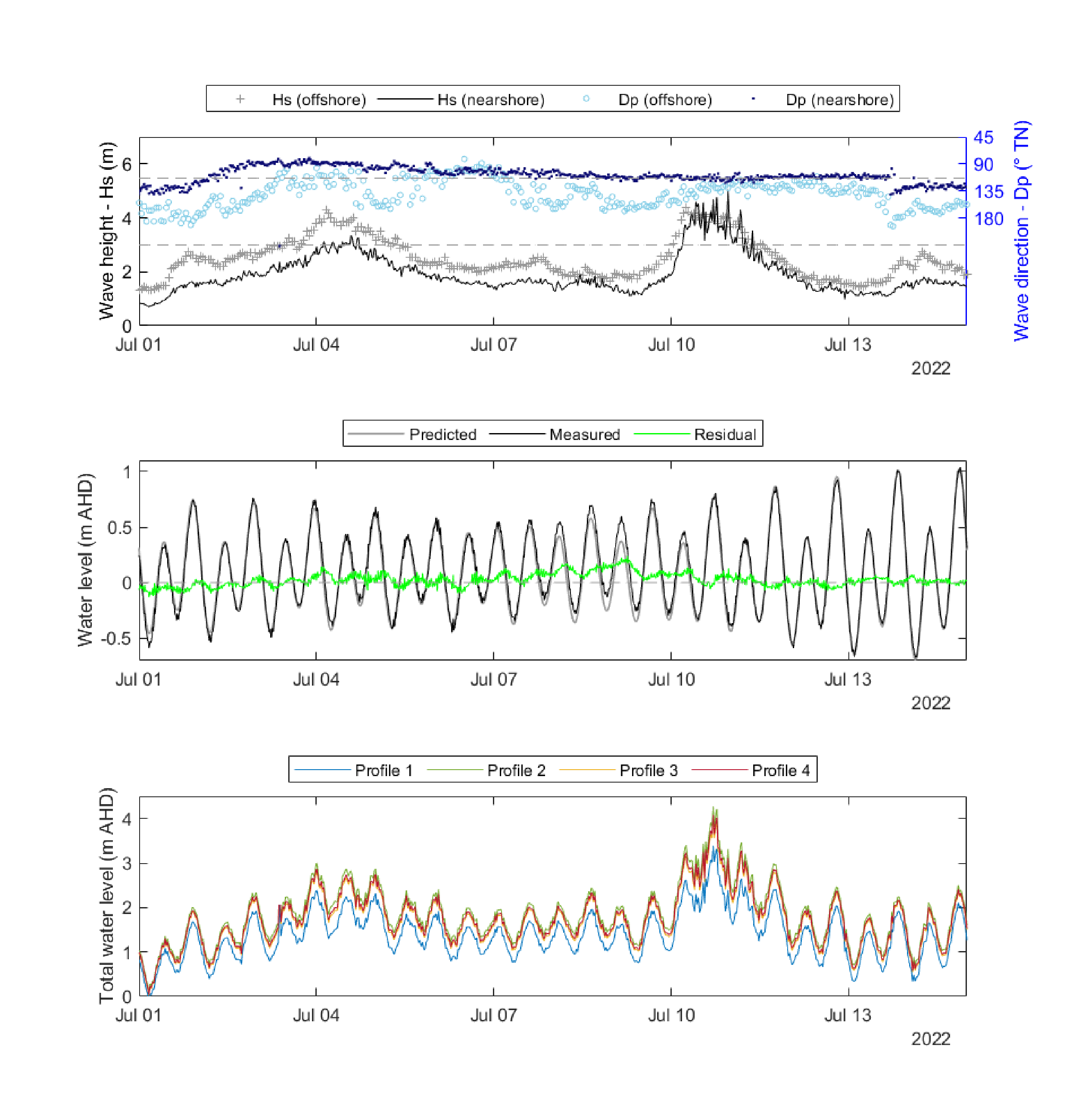
Supplementary Figure 3: Storm events of early July showing wave height (Hs = H_sig_) and direction perpendicular to Bengello Beach for the offshore wave buoy and the nearshore wave buoy at Bengello Beach. Water level data (predicted, measured and residual between these) from the same period is recorded by the nearby Princess Jetty tide gauge in Batemans Bay which represents the ocean tide with the additional influence of river floods. Total Water Level are shown for each of the four profiles.


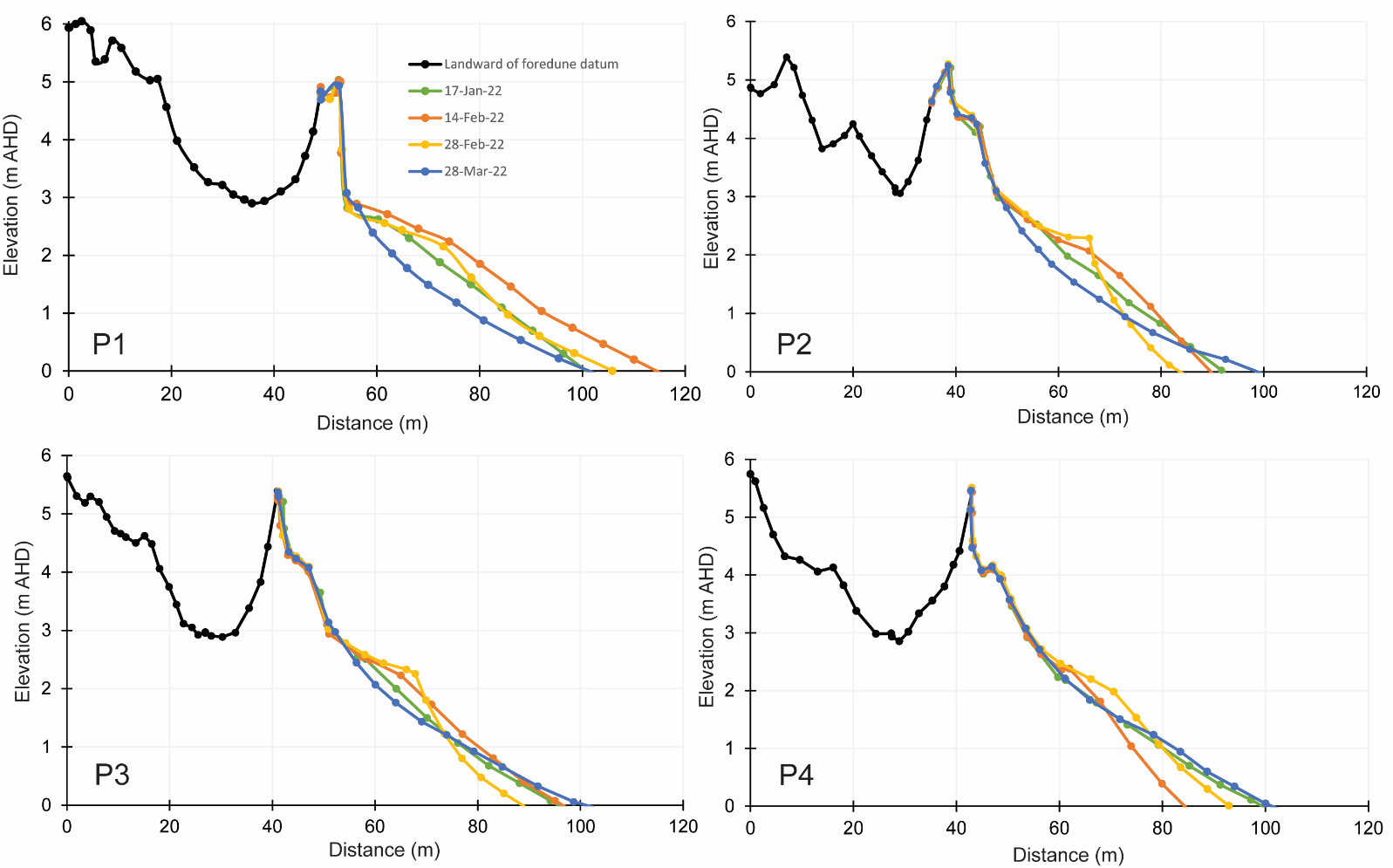


Supplementary Figure 4: Beach profiles from January to March 2022 showing the subtle berm feature which developed during February, but which was removed by storm events during early-mid March resulting in a concave profile seen in the survey on the 28^th^ March. This March survey occurred immediately prior to the storm from 31^st^ March – 4^th^ April (Supp Fig. 2) where wave overtopping of the foredune occurred with debris rafted into the swale and trimming back of the seaward margin of the foredune. The development of the concave profile geometry immediately prior to 31^st^ March – 4^th^ April storm likely amplified wave runup across the upper beach face.


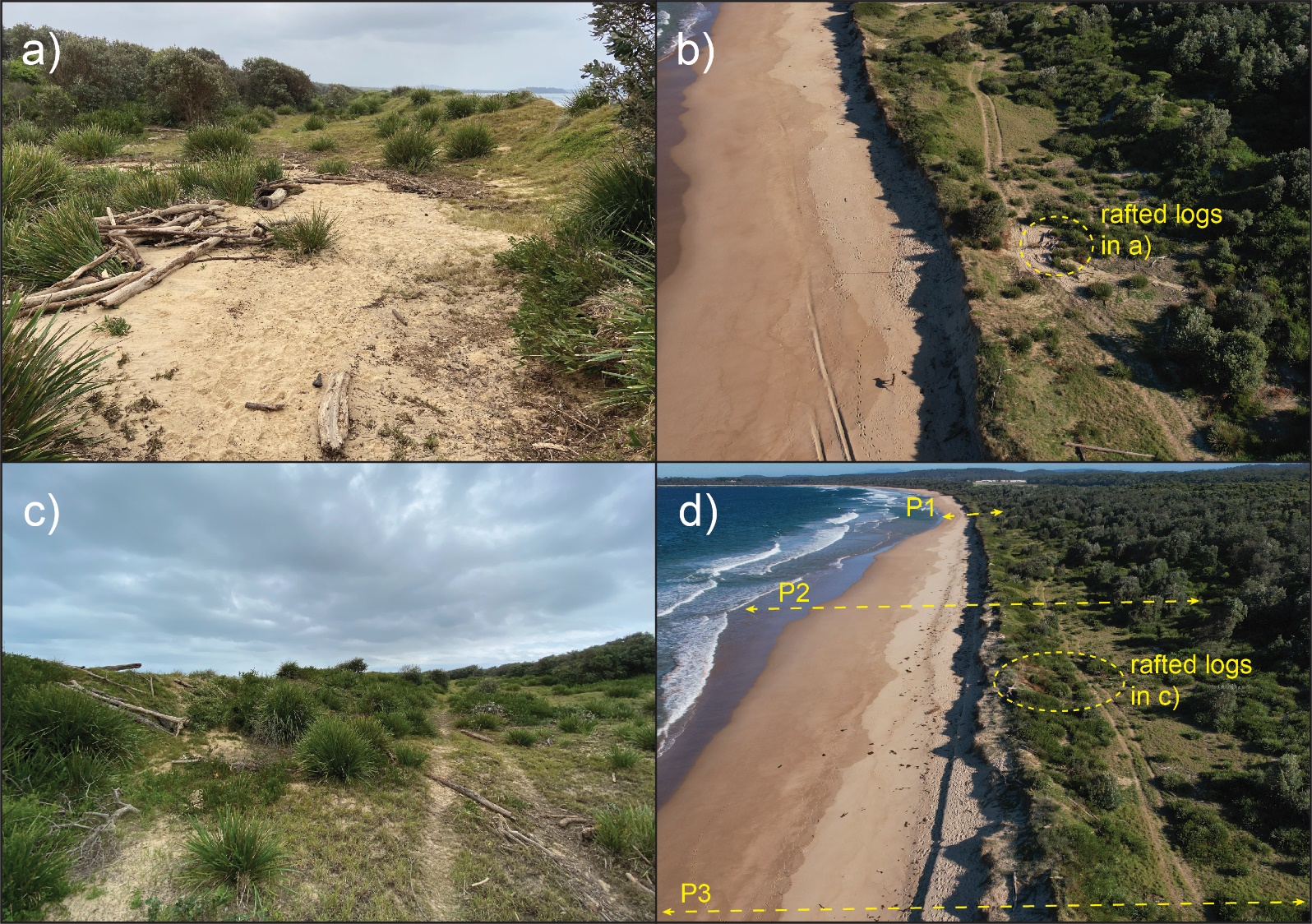


Supplementary Figure 5: Photos of debris rafter over the foredune during the storm event which peaked during the late evening of 1^st^ April and in the early hours of the 2^nd^ of April.


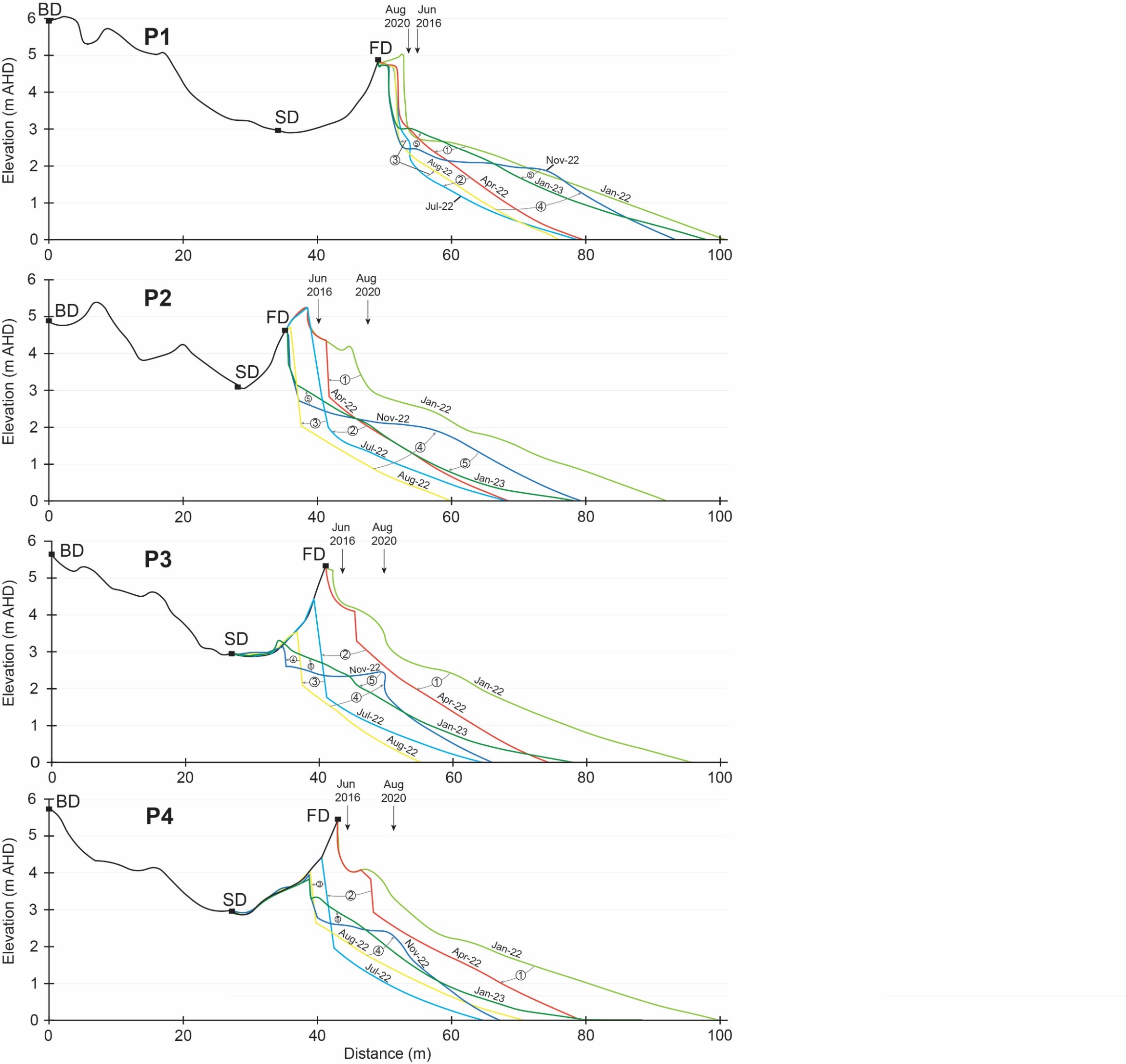


Supplementary Figure 6: Selected beach surveys from Profile 1 to 4 (P1 – P4) from 2022. Note that here, 0 m is the Back Datum (BD) whereas in the main manuscript, Figure 4 shows distance from the Swale Datum (SD). FD = Foredune Datum. Vertical black arrows indicate the position of scarps which developed as a result of the June 2016 storm events and the storms in Aug 2020.


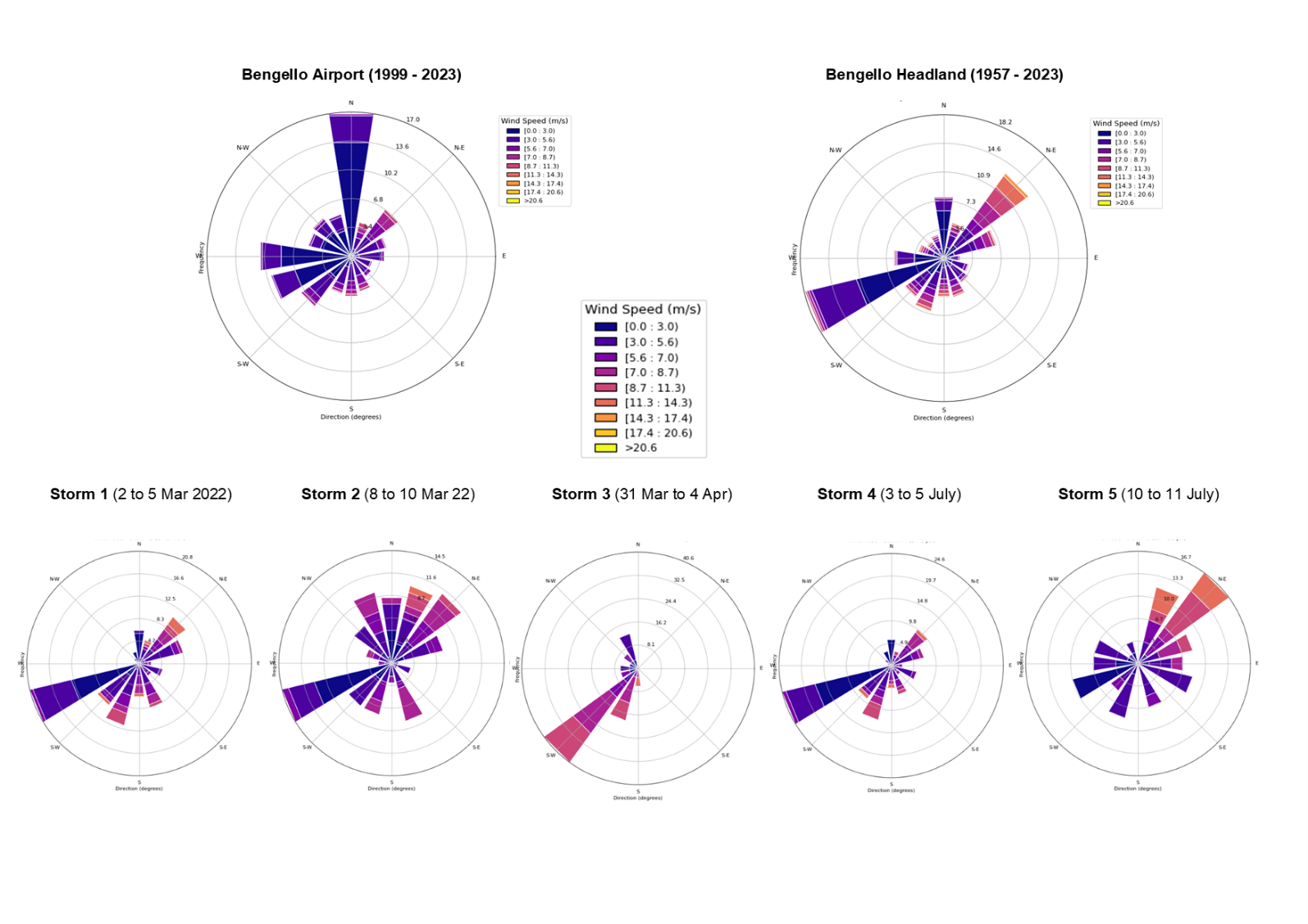


Supplementary Figure 7: Wind roses from Bengello Airport and Bengello Headland over their respective recording periods with accompanying wind roses for the five major storms recorded during the study period from the Moruya Heads station.


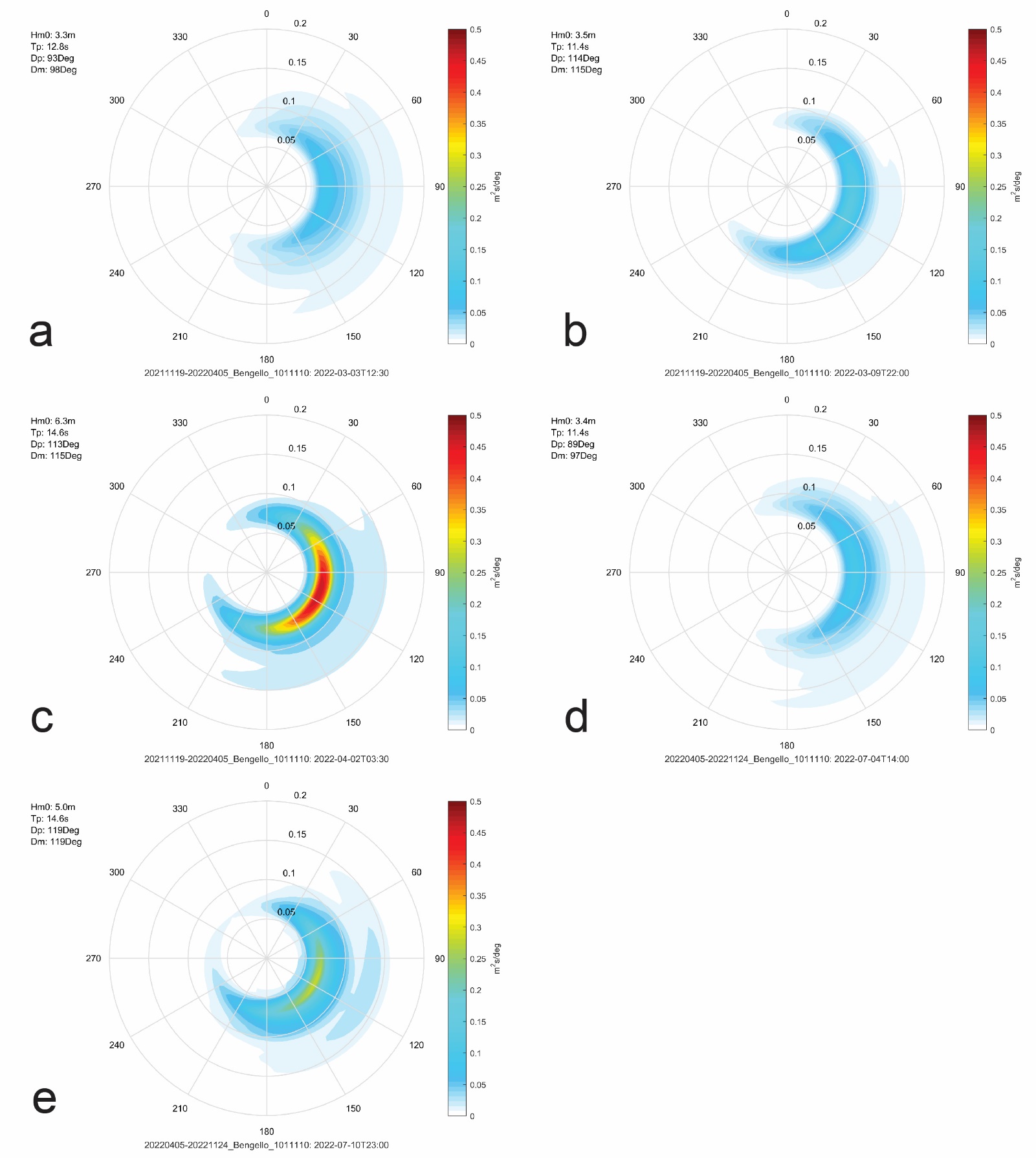


Supplementary Figure 8: Two dimensional wave spectra from the nearshore wave buoy positioned adjacent to Bengello Beach for the five major storm events in 2022. In these plots spectral significant wave height Hm0 (from zeroth-order moment of 1D energy spectrum) is H_sig_ and the peak wave period Tp is the period associated with the frequency at the peak of the energy spectrum, that is the frequency of highest energy density. Dp is the direction corresponding to the peak of wave energy while mean wave direction (Dm) averages direction across all wave frequencies present.

a) Peak recorded wave conditions during the first storm in March – ‘Storm 1’ (Supp. Fig. 1) showing a moderate-energy (H_sig_ = 3.3 m, Tp = 12.8 s) east swell (Dp = 91) with moderate-strong WSW winds with other variable directions throughout the storm producing some local sea wave growth (Supp. Fig. 7).

b) Peak recorded wave conditions during the second storm in March – ‘Storm 2’ (Supp. Fig. 1) showing a moderate-energy (H_sig_ = 3.5 m, Tp = 11.4 s) east-southeast swell (Dp = 111) with moderate-strong WSW winds with other variable directions throughout the storm producing some local sea wave growth (Supp. Fig. 7).

c) Peak recorded wave conditions during the storm in early April – ‘Storm 3’ (Supp. Fig. 2) showing a high-energy (H_sig_ = 6.3 m, Tp = 14.6 s) east-southeast swell (Dp = 114) with limited local sea wave growth due to strong offshore (SSW) winds at the storm peak and consistently throughout the storm (Supp. Fig. 7).

d) Peak recorded wave conditions during the storm in early July – ‘Storm 4’ (Supp. Fig. 3) showing a moderate-energy (H_sig_ = 3.4 m, Tp = 11.4 s) east swell (Dp = 97) with moderate-strong WSW winds with other variable directions throughout the storm producing some local sea wave growth (Supp. Fig. 7).

e) Peak recorded wave conditions during the storm in mid July – ‘Storm 5’ (Supp. Fig. 3) showing a high-energy (H_sig_ = 5 m, Tp = 14.6 s) east-southeast swell (Dp = 114) with strong local sea wave growth from strong onshore (NNE and NE) winds at the storm peak and with some variability throughout the storm (Supp. Fig. 7).


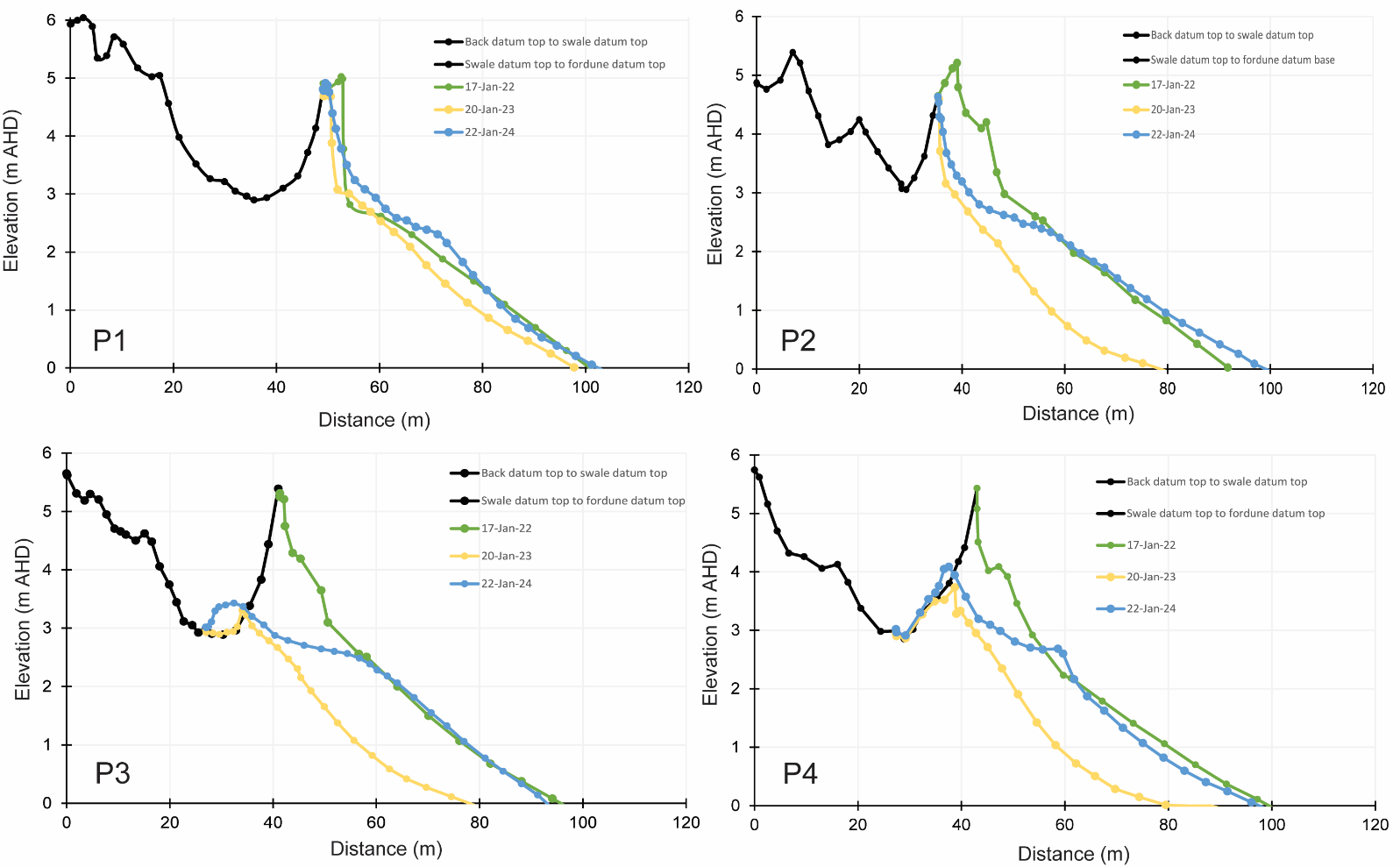


Supplementary Figure 9: Beach profiles from January 2022, January 2023, January 2024 showing the dramatic changes to Profile 3 (P3), the substantial changes to adjacent Profiles 2 and 4, and the relative stability of Profile 1 (P1).


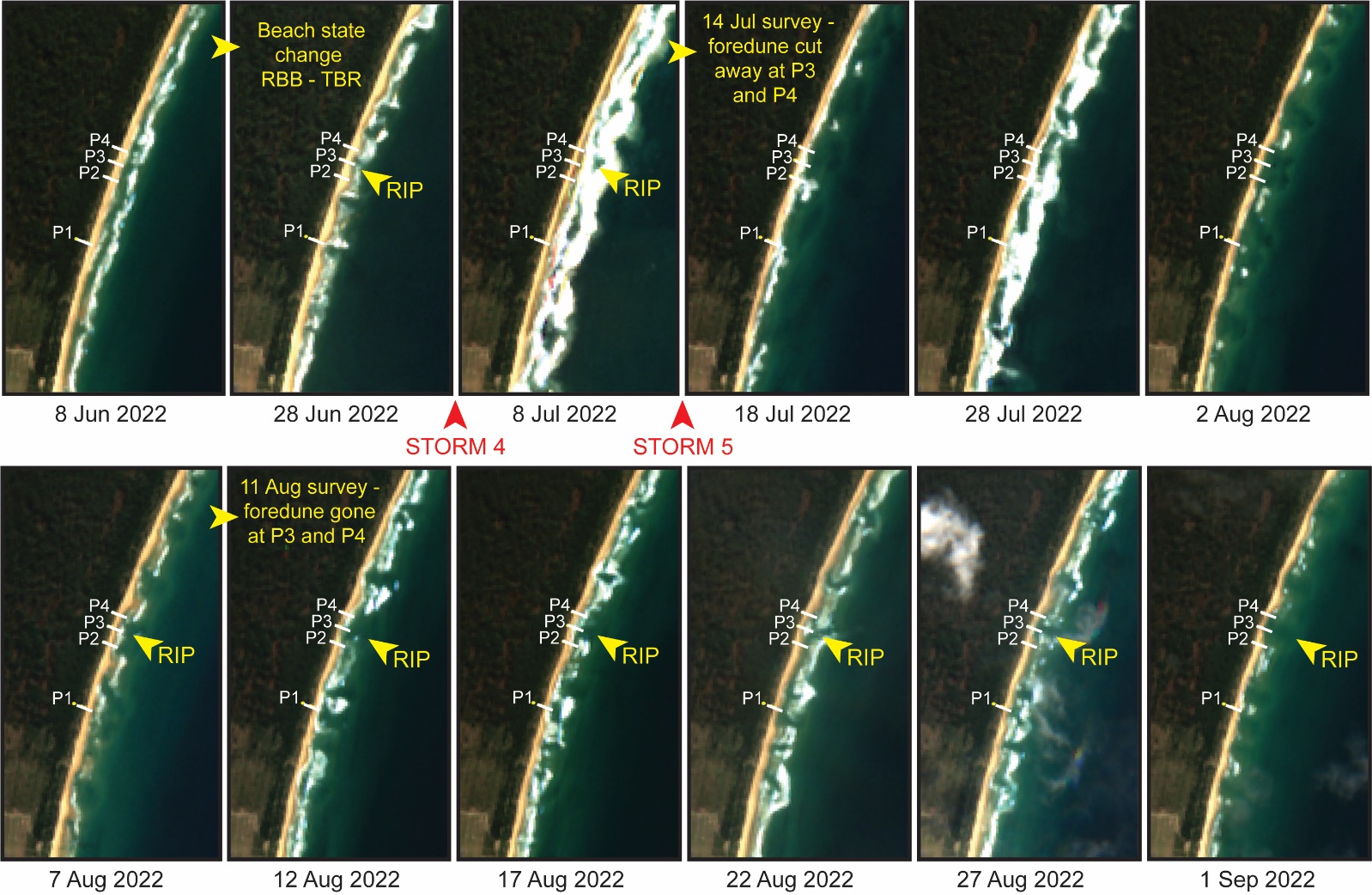


Supplementary Figure 10: A selection of cloud-free satellite images from Sentinel-2 for the period June 2022 to September 2022. A notable change in beach state occurred in June from Rhythmic Bar and Beach (RBB) to Transverse Bar and Rip (TBR) with rip embayments at Profile 3 and just south of Profile 1 developing immediately prior to Storm 4 (3-5 July) and Storm 5 (10-11 July). The beach and surfzone in the image on 20^th^ June also displays a megacusp morphology. Surf zone features from the image on the 8^th^ of July are difficult to decipher but it appears as though a rip embayment persisted and likely enabled wave attack of the foredune during Storm 5. It was on the 14^th^ July that we noted the foredune severely cut away at P3 and P4. The image from the 18^th^ of July shows how little beach remined in front of the foredune at this time at P3 compared with P1. Throughout the remainder of July and throughout August, the surfzone adjacent to P1 seems variable, while adjacent to P3 a rip embayment appears is evident in most image dates. We noted further removal of the foredune at P3 on the 11^th^ August when P3 achieved its minimum volume for 2022. Comparing the image dated 28^th^ June with those of August, it is notable that the crenulate pattern of the megacusps visible in June becomes mirrored in the foredune toe throughout August indicating variable foredune impact supporting the differences in profile behaviour of P1 and P3.


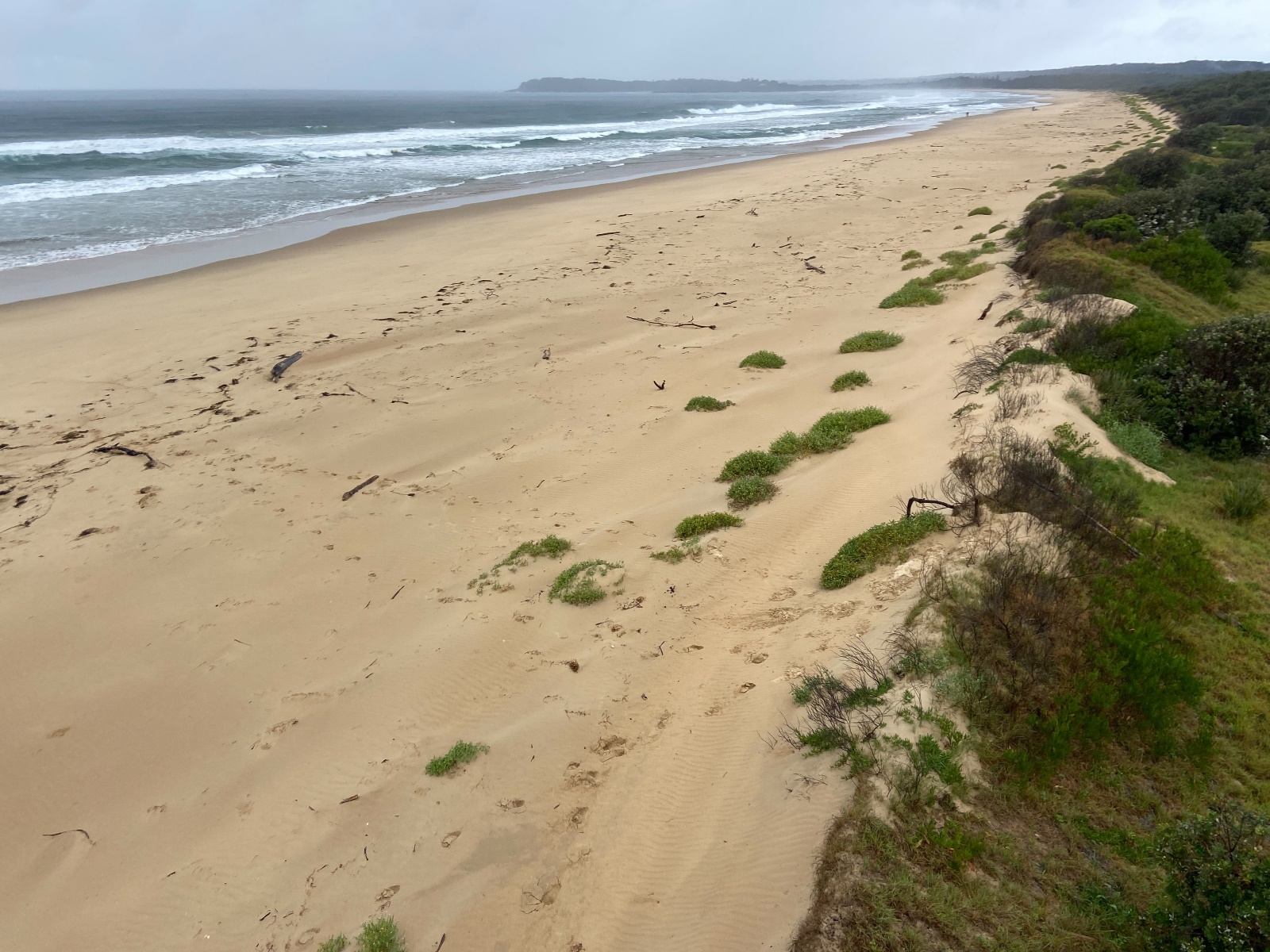


Supplementary Figure 11: Photo taken March 2024 looking south from Profile 2 showing crenulate due toe in the process of recovery via wind-blown sand and vegetation colonisation. This crenulate morphology was also evident in the foreshore in the aftermath of the July storms (see Supp. Fig. 10) and persisted for several months. Photo: T. Oliver.
